# Genome-wide comparisons of gene expression in adult versus elderly burn patients

**DOI:** 10.1371/journal.pone.0226425

**Published:** 2019-12-13

**Authors:** Stephanie C. Dreckmann, Saeid Amini-Nik, Ronald G. Tompkins, Miliana Vojvodic, Marc G. Jeschke

**Affiliations:** 1 Ross Tilley Burn Centre, Sunnybrook Health Sciences Centre, Department of Surgery, Toronto, ON, Canada; 2 Division of Plastic Surgery, University of Toronto, Toronto, ON, Canada; 3 Division of Surgery, Massachusetts General Hospital, Boston, MA, United States of America; 4 Harvard Medical School, Boston, MA, United States of America; University of Florida, UNITED STATES

## Abstract

**Purpose:**

Mortality and morbidity rates of elderly burn patients remain high despite numerous advancements in modern burn care. While prior studies have offered first insights on the biochemical changes in elderly burn patients compared to adults, the underlying cellular responses remain largely unknown. In this study, we aim to characterize the transcriptome of elderly burn patients and compare it to adult burn patients to obtain insights into the underlying molecular responses post-burn and to elucidate the effect of advanced age on the acute burn response.

**Materials and methods:**

Microarray data obtained from the Glue Grant Trauma-Related Database was obtained from blood specimens for ten elderly patients (n = 10), each with a set of two sex and total body surface area (TBSA) matched adult controls (n = 20), during the acute phase post-burn. Adult and elderly demographics and clinical outcomes were contrasted using using the Chi-Square test, Fisher’s Exact Test, or two-sample t-tests, as appropriate (p<0.05). Enrichment and heat maps were generated to compare gene expression in elderly versus adult burn patients.

**Results:**

Supervised analysis identified multiple genes that were differentially expressed between the elderly and adult groups. Pathway analysis and heatmap generation suggest that elderly patients share a distinct hypo-inflammatory response in the acute post-burn phase with downregulation of a number of immune-related pathways, including those related to antigen processing, specifically via MHC class I, ubiquitination and proteasome degradation (p<0.001, FDR < .001). Cell signalling pathways, such as NF-κB, C-type lectin receptor, and T cell receptor signalling were also significantly downregulated in elderly burn patients, as well as those relating to antiviral immunity (p<0.001, FDR < .001). Many genes which were observed to be upregulated in elderly patients with high TBSA burn injuries were associated with destruction-related cellular pathways such as complement activation and immunoglobulin production (p<0.005, FDR <0.01).

**Conclusions:**

The altered inflammatory and immune responses at the transcriptome level in elderly patients after burn are indicative of a failure in elderly burn patients to initiate an appropriate inflammatory and stress response during the acute phase post-burn.

## Introduction

Burns can be devastating injuries associated with high morbidity and mortality affecting nearly 500 000 individuals in the US alone[1]. Over the past 2–3 decades burn care providers made significant strides improving burn outcomes associated with implantation of burn intensive care units (ICUs), early surgical management, advances in local wound care, and an emphasis on nutrition[2,3]. Improvement in outcomes are reflected in a significantly increased LD50 (Lethal Dose 50, the total body surface area (TBSA) burn size associated with a 50% mortality risk) for pediatric and adult burn patients[3] from 45% in 1950[4] to 80% in 2015[1]. There is, however, one population where modern burn care has not affected outcomes; these are elderly (age ≥ 65) burn patients[5–7], whose LD50 remains at 25%[6]. Burn survival rates are far lower in elderly patients. A recent 20-year cohort study demonstrated an overall mortality of 7% in adult burn patients, compared to 29% in the elderly, despite similar burn size and inhalation injury rates. The discrepancy is even more significant in large burns (> 20% TBSA), with mortality rates of 24% versus 72%, respectively[5]. These abysmal results highlight the pressing need for improved elderly burn care.

It has been hypothesized that a failing immune system[8,9], multiple medical comorbidities[10], age-related skin changes[11], delayed wound healing[12], and altered cognition may contribute to poor elderly outcomes[5,6,13]. Another key variable in burn mortality is the hypermetabolic response incited by burn injuries leading to profound systemic catabolism. Elderly patients have a prolonged hypermetabolic response post-burn associated with increased glucose and lipid alterations[5,6]. This population also has a unique and detrimental inflammatory trajectory after burn. While adult patients have a hyperinflammatory response followed by recovery, the elderly have a hypo-inflammatory response followed by a hyperinflammatory response associated with immune dysfunction and most-likely exhaustion[5,6,9]. The adverse consequences of an augmented and prolonged hypermetabolic response are vast: catabolism, multi-system organ failure, and death. While prior studies have offered first insights on the biochemical changes in the elderly compared to adults, the underlying cellular responses remain largely unknown[5,9,14]. Transcriptome genomics allows for high-throughput screens of intracellular signaling pathways in the background of various clinical pathologies. In this study, we aim to characterize the transcriptome of elderly burn patients and compare it to adult burn patients to obtain insights into the underlying molecular responses post-burn and to elucidate the effect of advanced age on the acute burn response.

## Materials and methods

A large multi-centric study entitled *Inflammation and the Host Response to Injury* enrolled 573 burn patients to analyze genomic responses to trauma[15]. Subjects were enrolled between 2003 and 2010, with following inclusion criteria: admission to a participating centre within 96 hours post-burn, involvement of at least 20% total body surface area, and requirement of at least one surgical excision and skin grafting procedure. Patients were enrolled if they were expected to survive; futile patients were excluded.

Participating centres included institutions affiliated with University of Washington (Seattle, WA, USA), Massachusetts General Hospital (Boston, MA, USA), Loyola University Medical College (Chicago, IL, USA), University of Texas Medical Branch (Galveston, TX, USA), University of Texas Southwestern (Dallas, TX, USA), and University of Florida College of Medicine (Gainesville, FL, USA). Appropriate Institutional Review Board (IRB) approval was obtained from the Sunnybrook Research Ethics Board. Blood samples were obtained from each participant and were processed according to the protocols established and published by the Glue Grant[15]. Treatment of all enrolled patients was consistent with established standard of care protocols to minimize clinical treatment variation[16]. Demographics (age, gender, total body surface area) and in-hospital complications were prospectively recorded during acute admission. All collected data, including demographics and outcomes, are available through the Glue Grant Trauma-Related Database[15].

This study aimed to characterize the transcriptome of elderly burn patients utilizing the data collected in the aforementioned study. All elderly burn patients (defined as age 65 or older) with leukocyte microarray data available (Affymetrix U133 plus 2.0 array) from the acute phase post-burn (defined as the first 80 hours post-burn) were included. The Glue Grant microarray data can be found under accession number GSE11375 in the GEO DataSets. Each eligible elderly burn patient was matched with two appropriate burn size and gender-matched adult controls in the Glue Grant database. If two appropriate controls were not available, the patient was excluded from the study. If more than two controls were available, preference was given to younger patients to better outline potential age-related differences in gene expression. Elderly burn patient leukocyte microarray data were compared to adult controls and presented as outlined by Calvano et al.[17].

### Statistical analysis

Adult and elderly demographics and clinical outcomes were contrasted. Categorical factors were compared using the Chi-Square test or the Fisher’s Exact Test, as appropriate. Continuous variables were compared using two-sample t-tests. P values less than 0.05 were considered statistically significant.

### Enrichment maps

Enrichment maps were generated to compare gene expression in elderly versus adult burn patients. The gene expression data set (54675 probes) was preprocessed to remove probes that did not match any known gene and to collapse multiple probes matching a single gene. A single probe for each gene with the best t value from the moderated t-test was selected for subsequent analysis (20450 probes). The resulting data sets were analyzed using GSEA version 2.2.2.2 with parameters set to 2000 gene-set permutations and gene-sets size between 15 and 800[18]. The gene-sets included in the GSEA analyses were obtained from KEGG, NCI, Biocarta, Reactome and the Gene Ontology (GO, including IEA) databases, updated October 18, 2015. An enrichment map (version 2.0.1 of Enrichment Map software[19] in Cytoscape version 3.2.1) was generated for each comparison using enriched gene-sets with nominal p-value, false discovery rate (FDR) cut-off and overlap coefficient as indicated in each map and map descriptions. Differential comparisons between elderly and adult control arrays were completed based on TBSA: all subjects with TBSA adjusted as a continuous variable (group 1), subjects with high TBSA burns (group 2, >33%), and subjects with low TBSA burns (group 3, ≤33%). Unless otherwise indicated, the nominal p values were corrected using the Benjamini-Hochberg procedure. Enrichment maps were filtered to contain genes related to immune system function, defined as descendants of the Gene Ontology “immune system process”, immune-related KEGG pathways, and descendants of the term “immune system” in Reactome[20–22].

### Heatmap generation

A string-matching search was performed on the gene-set output from GSEA for each of the comparisons using the terms: histiocyte, leukocyte, neutrophil, monocyte, macrophage, B cell, T cell, immune system, cytokine, chemokine, antigen. The resulting lists were ordered by increasing FDR. To generate the heatmaps, the significance threshold was set at 0.25 FDR for the gene-set level statistics for all three comparisons; the genes in the gene-sets were further filtered by gene-level FDR (FDR < 0.2 for low TBSA and linear TBSA; FDR < 0.25 for the high TBSA comparison), and then ranked according to their absolute log2FC. The expression values for the 50 top-ranking immune-related genes were then plotted (gplots v:3.0.1, with complete hierarchical clustering and the Pearson correlation as distance) for each subgroup. Expression values (log2) were Z-normalized across rows.

## Results

### Patient demographics

Of the 573 enrolled burn patients in the Glue Grant Trauma-Related Database, 27 were defined as elderly (age ≥65). Twelve of these 27 patients had leukocyte microarray data available for analysis. Two elderly patients did not have burn size and gender-matched controls and hence were excluded. A total of 10 elderly burn patients were analyzed, each with two corresponding adult controls. Demographics for the 30 examined burn patients are shown in Table 1.

**Table 1:** Demographics.

Demographics	All patients n = 30	Patients 18–64 years n = 20	Patients ≥65 years n = 10	p-value comparing 18–64 vs. ≥65
Age years (Median)	42 ± 22 (34)	28 ± 7 (27)	72 ± 7 (69)	< .0001
Gender M [n (%)]	24 (80)	16 (80)	8 (80)	1.00
Burn TBSA (%) (Median)	37 ± 14 (34)	37 ± 14 (34)	37 ± 14 (35)	0.99
Inhalation Injury [n (%)]	9 (30)	5 (25)	4 (40)	0.42
**Burn Mechanism**				1.00
Flame n (%)	23 (77)	15 (75)	8 (80)	
Flash n (%)	5 (17)	4 (20)	1 (10)	
Scald n (%)	2 (7)	1 (5)	1 (10)	
**Ethnicity**				1.00
Caucasian n	23 (77)	15 (75)	8 (80)	
Hispanic n	2 (7)	2 (10)	0	
African American n	3 (10)	2 (10)	1 (10)	
Others n	2 (7)	1 (5)	1 (10)	

Demographics for all subjects, adults (18–64 years), and elderly (≥65 years). TBSA = total body surface area. Data presented as mean ± SD or frequencies.

The mean age of adult patients and elderly patients was 28±7 years, and 72±7 years, respectively. The mean TBSA for adult and elderly patients was identical in both groups: 37 ± 14%. Inhalation injury was observed in 25% of adult patients, compared to 40% of elderly patients (p = 0.42). Flame, flash, and scald burns were documented as the underlying mechanism in 75%, 20%, and 5% of the adult patients, compared to 80%, 10% and 10% of the elderly patients, respectively (p = 1.00). Comparisons in clinical outcomes for the examined adult and elderly burn patients are outlined in Table 2. Average hospital length of stay for burn survivors (deaths were excluded) was 45±37 days for the adult group and 39±29 for the elderly group (p = 0.77). Burn wound infection developed in 55% of adult patients, compared to 50% in the elderly group (p = 1.00). Nosocomial infection was observed in 65% of adult patients and 90% of elderly burn patients (p = 0.21). Survival rates were significantly higher in the adult group (95%), compared to the elderly group (50%, p = 0.009).

**Table 2:** Patient outcomes.

Characteristics	All patients n = 30	Patients 18–64 years n = 20	Patients ≥65 years n = 10	p- value comparing 18–64 vs. ≥65
Length of Hospital Stay (days) Median (IQR)	44 ± 35 32 (18–60)	45 ± 37 32 (18–61)	39 ± 29 32 (25–44)	0.77
Length of Hospital Stay (days)/ TBSA% Median (IQR)	1.2 ± 0.72 1.0 (0.73–1.52)	1.2 ± 0.7 1.0 (0.70–1.44)	1.3 ± 0.7 1.5 (0.78–1.83)	0.63
Denver 2 Score Median (IQR)	3 ± 2 2 (0–3)	2 ± 2 2 (0–3)	4 ± 3 3 (2–5)	0.076
Burn wound infection (Y/N) n (%)	16 (53)	11 (55)	5 (50)	1.00
Nosocomial Infection n (%)	22 (73)	13 (65)	9 (90)	0.21
Mortality n (%)	6 (20)	1 (5)	5 (50)	0.009

Clinical outcome parameters for all subjects, adults (18–64 years), and elderly (≥65 years). TBSA = total body surface area. Data presented as means ± SD or frequencies.

### Enrichment maps

#### Group 1 (TBSA as a linear covariate)

**Fig 1:**
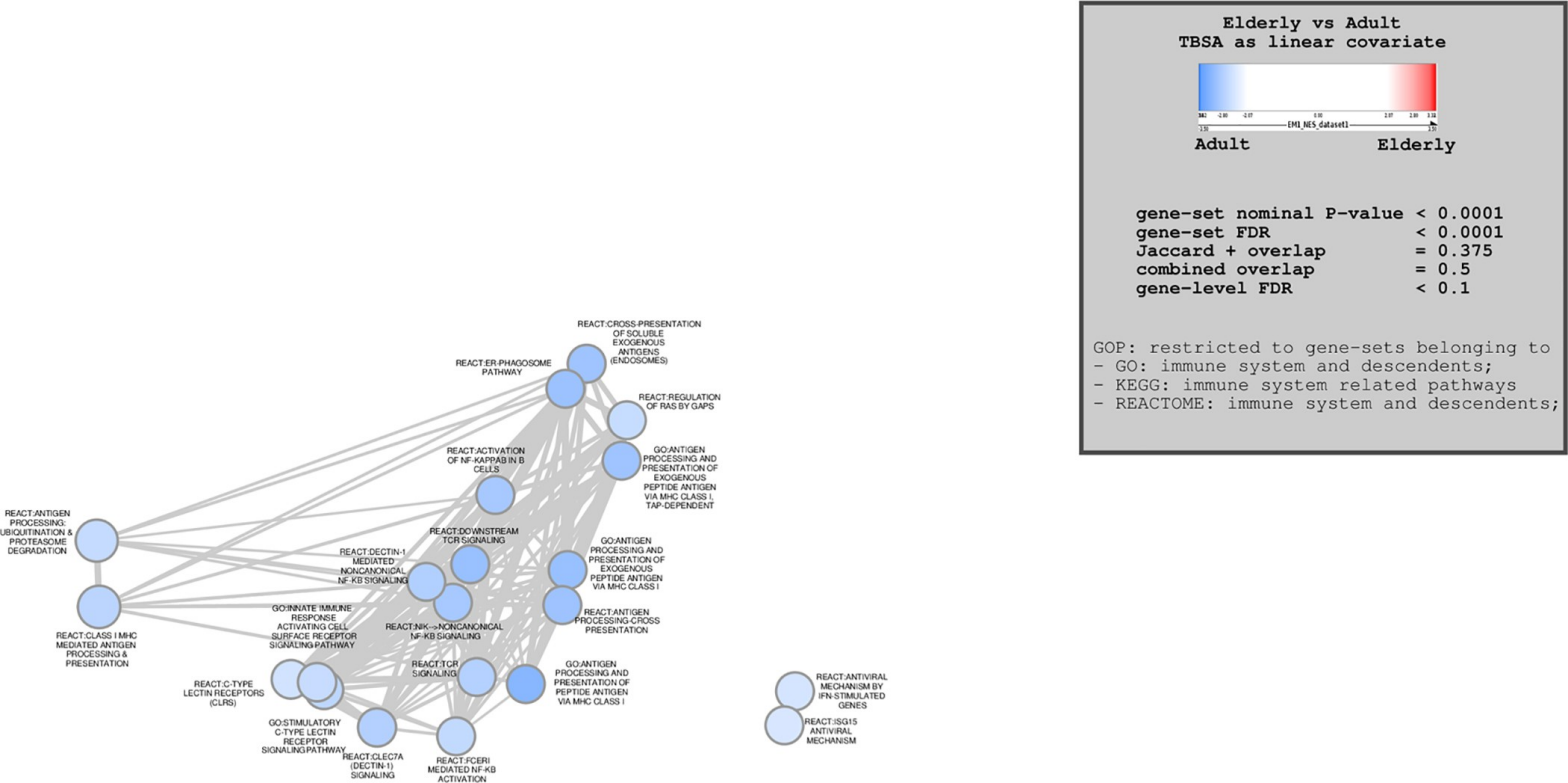
Enrichment map outlining significantly altered immune-related pathways for comparison group 1 (TBSA as a linear covariate). Blue nodes represent pathways downregulated in the elderly group, red nodes represent pathways upregulated in the elderly group. Node size is proportional to gene set size.

Of 7490 examined gene sets, 4033 were upregulated in the elderly group and 3457 were downregulated when directly contrasted with adult controls (p<1). When a p-value of 0.0001 was utilized to detect statistically significant differences, 888 genes were significantly upregulated in the elderly group and 1451 were downregulated. Enrichment maps revealed multiple immune-related pathways were found to be downregulated in elderly patients compared to adults (Fig 1). Elderly patients in this subgroup showed altered expression of genes related to antigen processing, specifically via MHC class I, ubiquitination and proteasome degradation. Many pathways involved in cell signalling, such as NF-κB, C-type lectin receptor, and T cell receptor signalling were significantly downregulated. Pathways relating to antiviral immunity were also suppressed in the elderly.

#### Group 2 (High TBSA)

**Fig 2:**
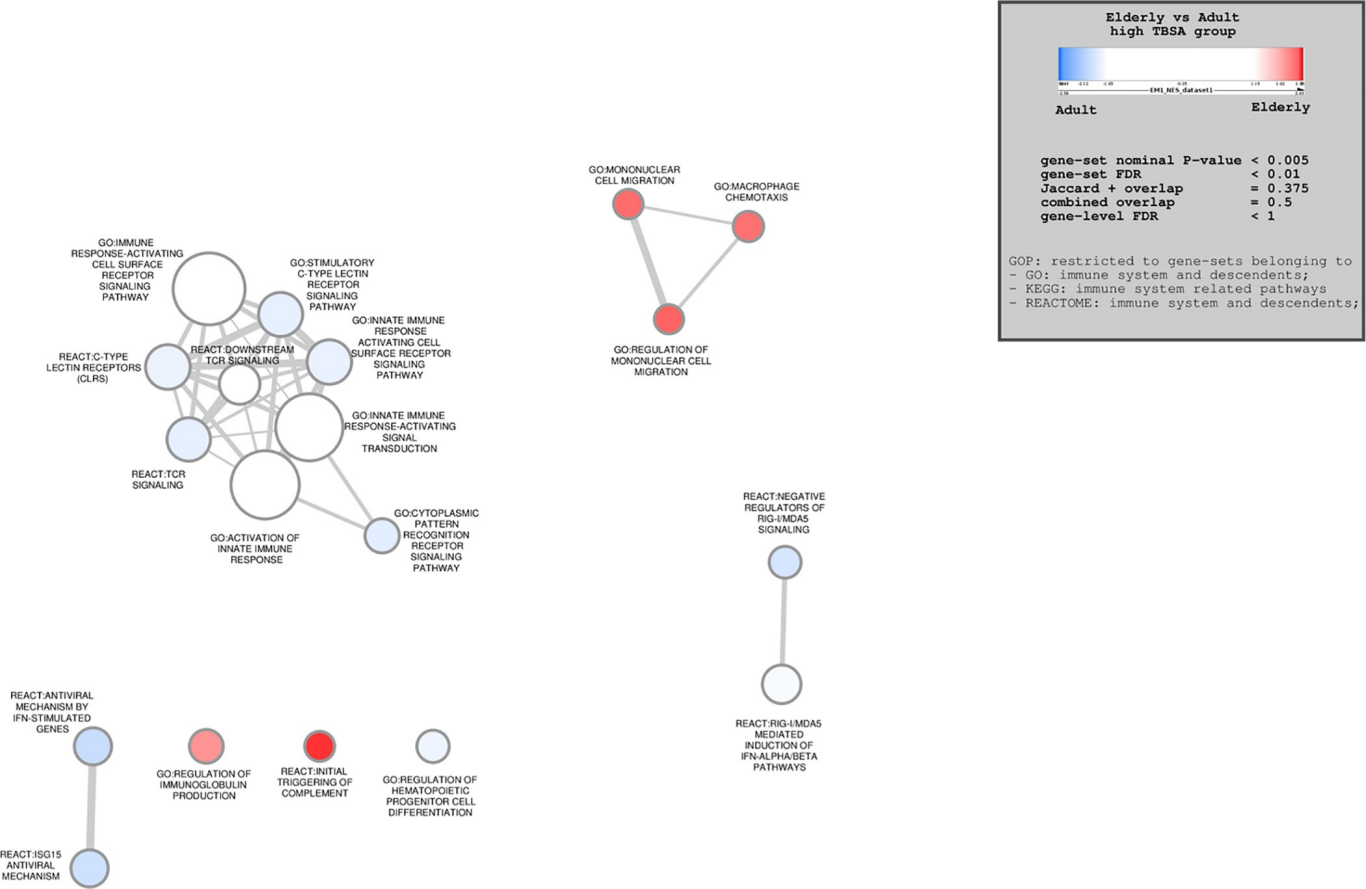
Enrichment map outlining significantly altered immune-related pathways for comparison group 2 (high TBSA). Blue nodes represent pathways downregulated in the elderly group, red nodes represent pathways upregulated in the elderly group. Node size is proportional to gene set size.

Of the 7488 gene sets examined, 5114 were upregulated in the elderly group, and 2374 were downregulated compared to adult controls (p<1). When a p value of 0.0001 was utilized to detect statistically significant differences, 621 genes were significantly upregulated in the elderly group, while 512 were significantly downregulated. As TBSA increased, more upregulated pathways in elderly patients were detected, however, the global picture was that the transcripteomic profile in elderly burn patients is profoundly downregulated (Fig 2). Once again, multiple pathways involved in cell signalling, such as C-type lectin receptor, immune response-activating cell surface receptor and T cell receptor signalling were significantly downregulated. Pathways responsible for the activation of the innate immune response were downregulated in the elderly group. Downregulation of genes related to antigen processing and antiviral immunity was once again observed in the elderly. In this subgroup exclusively, elderly patients showed upregulation of destruction-related pathways, such as complement activation and immunoglobulin production.

#### Group 3 (Low TBSA)

**Fig 3:**
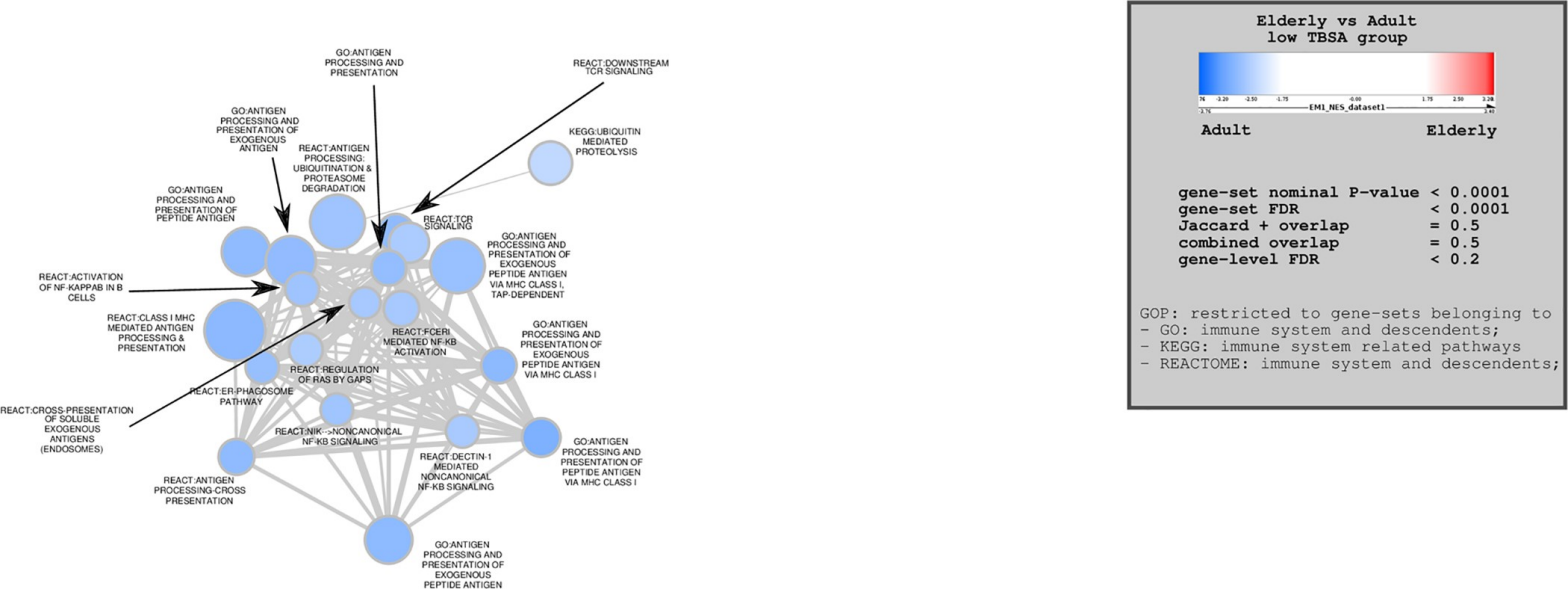
Enrichment map outlining significantly altered immune-related pathways for comparison group 3 (low TBSA). Blue nodes represent pathways downregulated in the elderly group, red nodes represent pathways upregulated in the elderly group. Node size is proportional to gene set size.

Of the 7489 gene sets examined, 3124 were upregulated in the elderly group and 4365 were downregulated compared to adult controls (p<1). When a p value of 0.0001 was utilized to detect statistically significant differences, 826 genes were significantly upregulated in the elderly group, while 1859 were significantly downregulated. Elderly patients included in the low TBSA comparison group confirmed a picture consistent with a hypo-inflammatory response (Fig 3). No pathways were determined to be significantly upregulated in elderly patients. In contrast, several pathways related to antigen processing and cell-signaling (T cell receptor and NF-κB signalling, etc.) were once again confirmed to be downregulated in this subgroup. For additional information on which specific genes were upregulated or downregulated for each comparison group, consult supplementary tables 1 through 5 (S1, S2, S3, S4 and S5 Tables).

### Heatmaps

**Fig 4:**
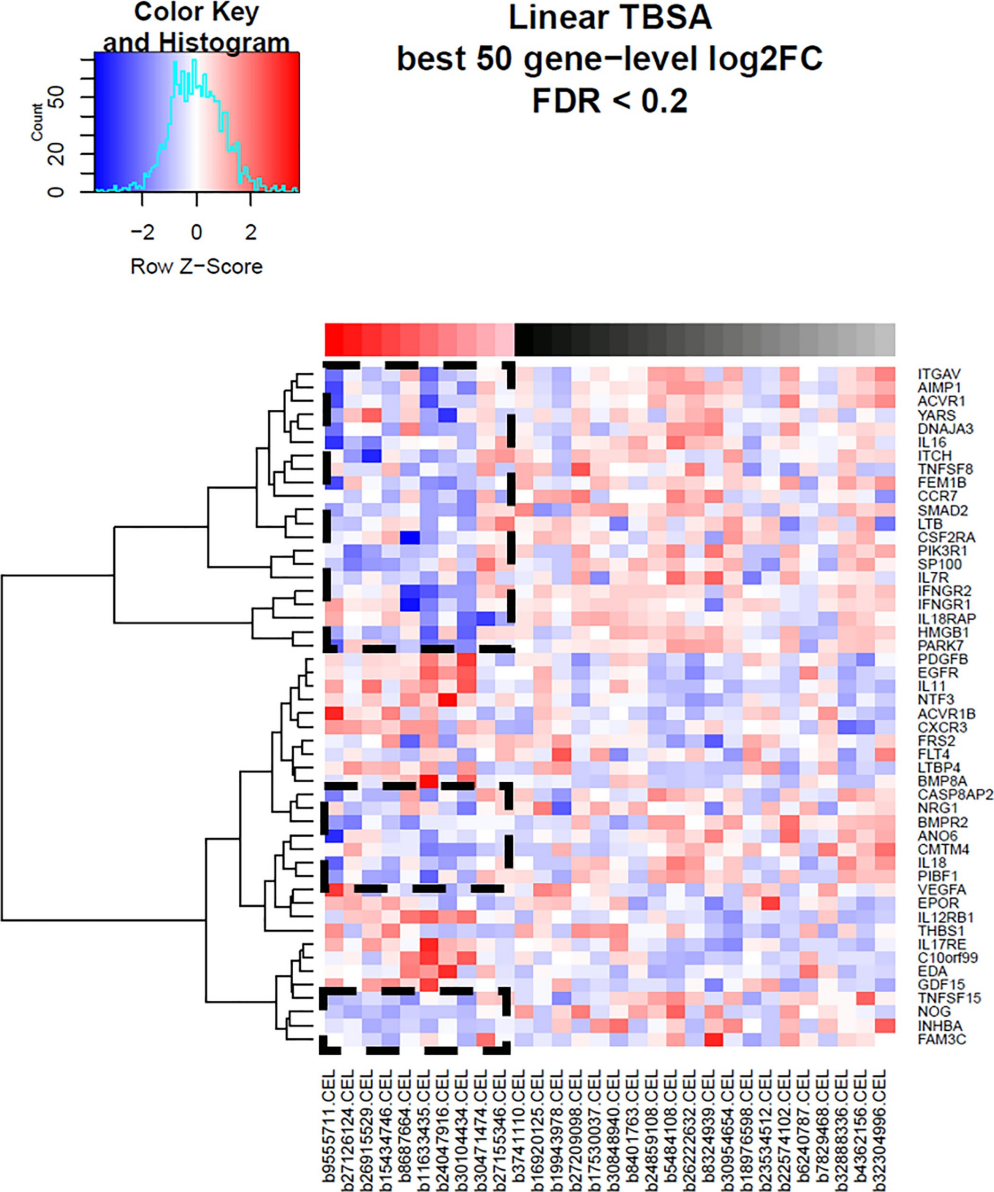
Heat map for comparison group 1 (TBSA as a linear covariate). Generated from hierarchical clustering of differentially expressed immune-related genes. The top colour blocks identify the sample type; red = elderly, black = adult, and shading corresponds to TBSA values. The color key outlines the degree of expression for each listed. gene. Boxed areas highlight downregulated genes in the elderly group.

**Fig 5:**
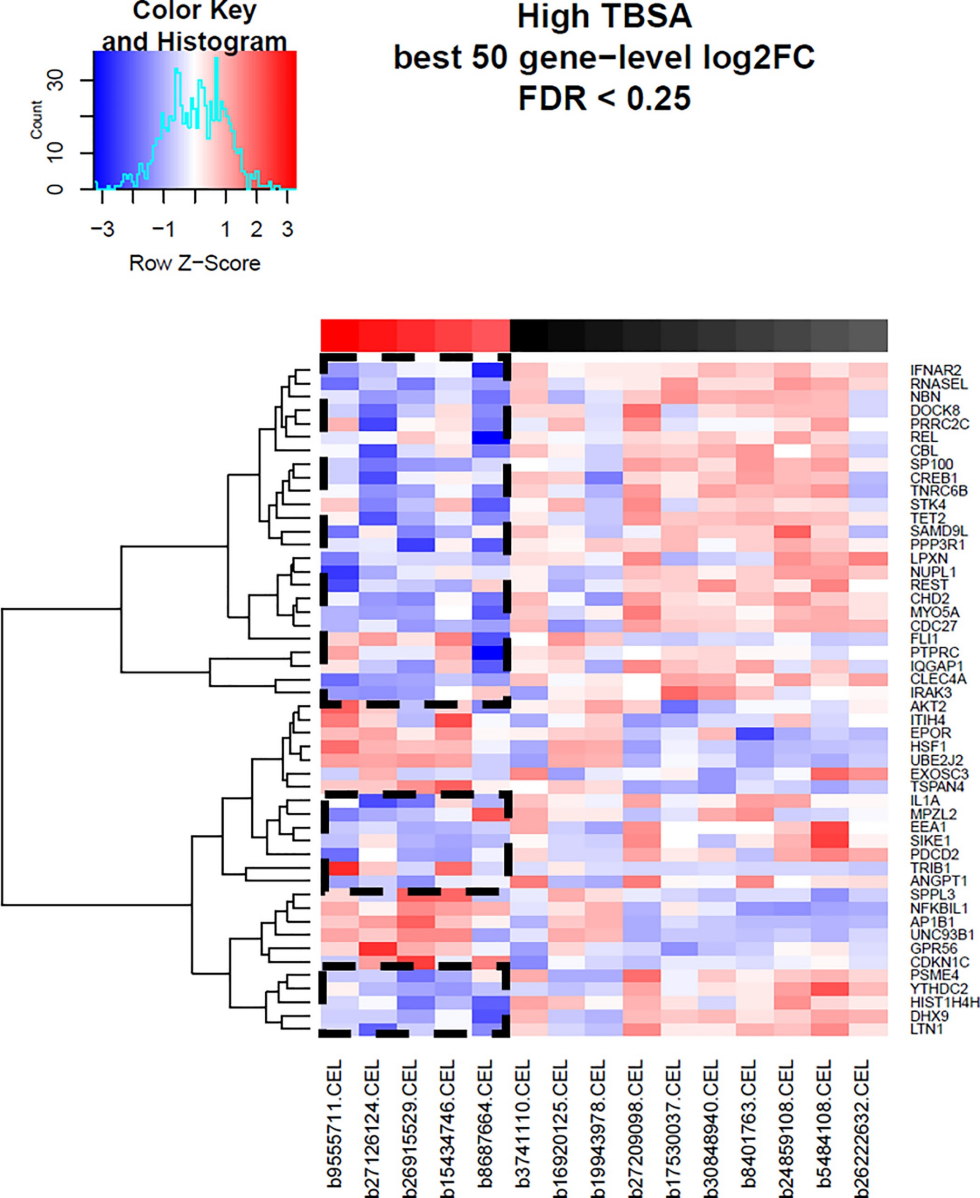
Heat map for comparison group 2 (high TBSA). Generated from hierarchical clustering of differentially expressed immune-related genes. The top colour blocks identify the sample type; red = elderly, black = adult, and shading corresponds to TBSA values. The color key outlines the degree of expression for each listed. Gene. Boxed areas highlight downregulated genes in the elderly group.

**Fig 6:**
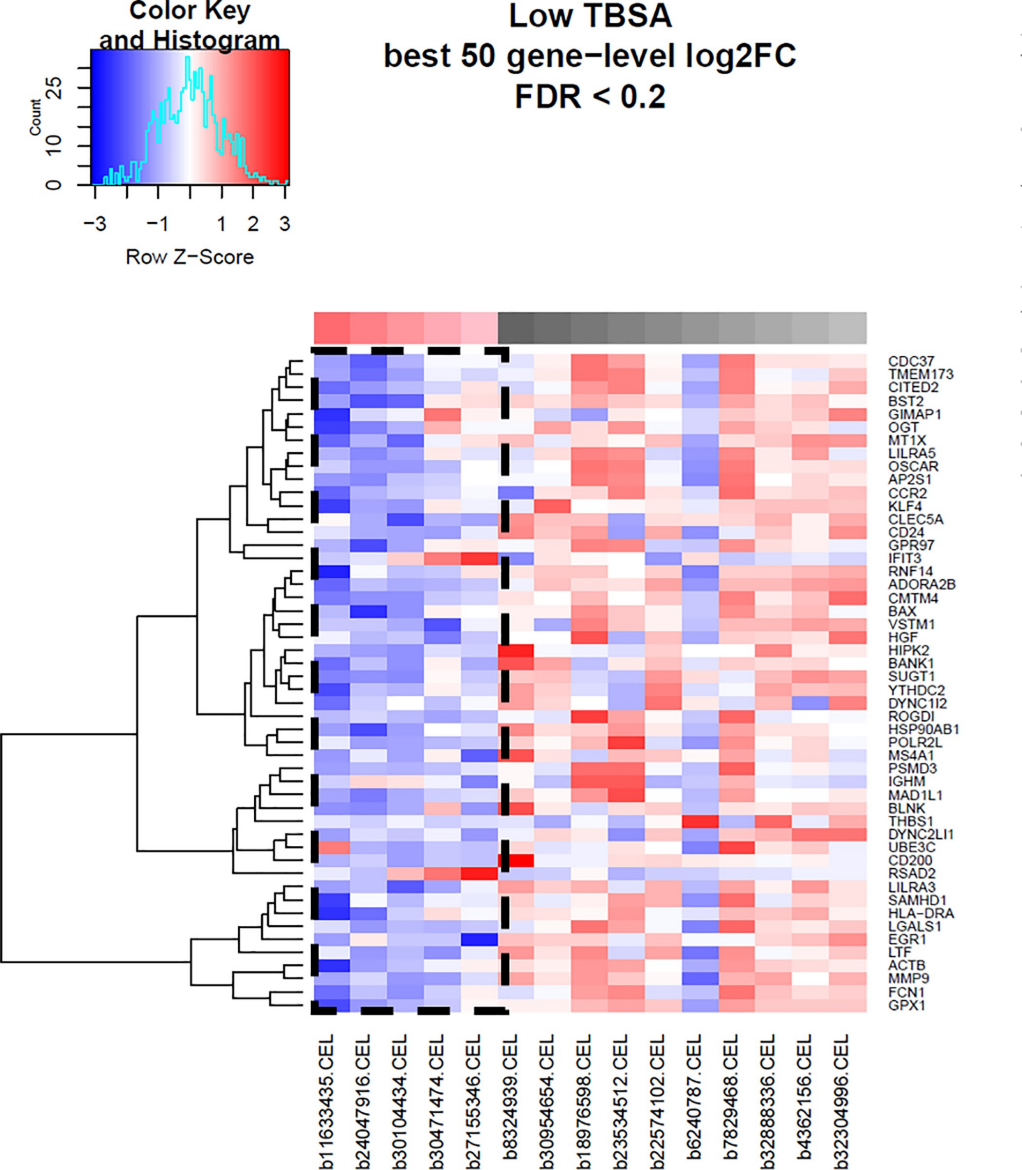
Heat map for comparison group 3 (low TBSA). Generated from hierarchical clustering of differentially expressed immune-related genes. The top colour blocks identify the sample type; red = elderly, black = adult, and shading corresponds to TBSA values. The color key outlines the degree of expression for each listed. gene. Boxed areas highlight downregulated genes in the elderly group.

Heat maps were generated for each comparison group using methods previously outlined (Figs 4–6, respectively). The top 50 hierarchically clustered, differentially-expressed immune-related genes for each comparison group are depicted along the y axis. Genes which were downregulated are depicted in blue, with darker colours representative of greater downregulation. Upregulated genes are depicted in red, with darker shading corresponding to a greater degree of expression. Across all three comparison groups, the majority of the depicted immune-related genes are downregulated in the elderly group. These include genes involved in cell signalling, angiogenesis, inflammation, wound healing, cytokine expression, lymphocyte activation and antiviral immunity. In elderly patients with low TBSA burns, nearly all immune-related genes are downregulated (Fig 6). With greater TBSA involvement, a minority of genes in the elderly group are upregulated (Figs 4 and 5). The profile of these genes vary greatly from the downregulated group and many are linked to the initiation of cell death.

## Discussion

We recently reported that elderly patients have an altered immune response to burns, characterized as a hypo- then hyper-inflammatory response[5]. The purpose of this study was to investigate the elderly burn patient transcriptome during the acute phase post-burn, and provide further insight into the specific pathways and genes which are altered during this period. Pathway analysis and heatmap generation suggest that elderly patients share a distinct hypo-inflammatory response in the acute post-burn phase with downregulation of a number of immune-related pathways, many revolving around cell signaling and antigen processing. This was consistent across all 3 comparison groups.

With greater TBSA involvement, a minority of genes in the elderly group were upregulated (Fig 5) compared to adult controls. A number of the upregulated genes were involved in heat shock protein synthesis (HSF1) and protein degradation targeting (UBE2J2). Enrichment maps revealed similar findings (Fig 2): upregulated pathways in elderly patients with high TBSA burns were involved in hemolysis and complement activation. These findings may suggest that under stress, elderly patients have greater activation of destruction-related genes and pathways and may contribute to the poorer outcomes observed in elderly burn patients. Whether this observation is a cause for the poor outcome or it is the effect of other morbidities in the elderly cannot not be addressed with this study as the n is not enough to obtain sufficient power. It is imperative, however, to conduct studies to determine underlying physiologic and cellular mechanisms on why the elderly have a significantly higher mortality.

Supporting our hypothesis that genomic or epigenomic changes in the elderly contribute to poor outcomes is a recent study from our group which demonstrated that elderly burn patients have a distinct acute phase burn response[14]. Elderly patients showed a compromised stress response including attenuated cardiac function and subsequently, organ perfusion. The elderly have lower inflammatory markers along with inadequate pathophysiologic responses that are required to survive[14]. We now add to the growing body of evidence that elderly burn patients are unable to respond to stress based on the transcripteomic profile. In this study we found that the majority of genes are downregulated rather than upregulated when compared to like-size burns that are younger, indicating that the failure to respond may derive from the genome.

Several genes related to erythroblast regulation and proliferation were also upregulated in the elderly group (EPOR, FLI1). It has been well-characterized that extensive thermal injury results in a significant and refractory anemia and the upregulation of these pathways in the elderly suggest that there may be a signal towards increased acute hemolysis of heat-damaged erythrocytes[23]. A few transcriptomes related to mononuclear cell migration, macrophage chemotaxis, immunoglobulin production, and complement activation were also upregulated in elderly high TBSA patients. This suggests that prolonged hyperinflammation in elderly burn patients might directly affect the expression of these genes.

Our study highlights significant downregulation of the immune response in elderly burn patients. In contrast, Sood et al. analyzed transcriptomic changes associated with burn age, size, and the presence of inhalation injury and established that there were no differentially expressed genes for burn patients aged >60. They do, however, comment that their study was possibly underpowered as only 12% of patients in their analysis were older than 60 and that further studies are needed in this group[24]. Another transcriptomic analysis demonstrated that burn patients hypersusceptible to infection demonstrated suppression of immune cell activation and early alterations in immune-related signaling pathways[25], similar to our findings in elderly patients. A murine analysis of the circulating leukocyte response post burn revealed upregulation of multiple cell signaling pathways which may be critical in initiating an appropriate immune response[26]. Our data confirmed that there is a downregulation of multiple cell signaling pathways in elderly burn patients, possibly negatively influencing clinical outcomes. A prior study compared cytokine responses between young and elderly burn patients. They elucidated that at least 2 cytokines, CCL5 and EGF, are downregulated in elderly burn patients[27]. Consistent with this finding, we report here that five genes involved in EGF signaling (associated with wound healing) are significantly downregulated in high TBSA elderly burn patients (S2 Table).

Focusing on specific pathways, our data demonstrate that NF-κB related pathways are significantly downregulated in the elderly burn group. NF-κB plays an integral role in regulating the immune response and has been linked to the progression of a number of diseases[28]. It is typically activated during periods of stress and has a number of immune-related effects, including the expression of proinflammatory cytokines and suppression of apoptosis[29]. This could possibly contribute to hypoinflammation as well as increased apoptosis and cell destruction.

Aging is a complex phenomenon associated with several cellular and molecular changes. While several mechanisms contribute to these changes, a complete understanding of the effects aging has on the human transcriptome has yet to be verified. There is an increasing body of evidence to suggest that aging is a product of multiple epigenomic changes, which alter in response to external factors such as environmental stimuli and nutrients[30]. Boule and Kovacs highlighted that alcohol consumption in elderly patients was associated with weakened initiation of immunity. Similar pathways as those demonstrated in our study, such as NF-κB, were downregulated in elderly patients who consumed alcohol[31]. Alcohol consumption is a significant risk factor for burn injury[32], and if combined with older age, there may be an exponential decrease in a patient’s ability to mount an appropriate immune response post burn. Our results revealed multiple additional downregulated pathways in elderly patients, including ubiquitination and proteasome degradation. This could result in epigenomic changes that contribute to the significantly altered response observed between elderly and adult burn patients. Overall, our data confirm that there is an altered, hypo-inflammatory immune response at the transcriptome level in elderly burn patients compared to adults. The products of these transcripts have an essential role in an efficient immune response, a phenotype that is deficient in elderly patients.

We would like to indicate the limitations of this study. We acknowledge that we only examined gene expression during the acute phase post burn, representing a single, early time point rather than expression over a longer time course. The expression profile will most likely change over time and with the current results, we have obtained only a small glimpse into the complex transcripteomic response of elderly burn patients. Elderly burn patients often tend to deteriorate later on during their hospital admission, rather than immediately following burn injury. Whether this delayed failure results from this initial diminished inflammatory response, or occurs in spite of it, is unclear and requires further study. Prior literature has suggested, however, that although it may become clinically apparent later on, this deterioration begins soon after injury and manifests in a multitude of ways, including cardiac depression and hypoinflammation[14].

The study is also limited by the relative underrepresentation of patients over the age of 65 in the host dataset. There are additional factors that may contribute to the differences observed in gene expression between adult and elderly burn patients, such as pre-existing comorbidities or inhalation injury. Although not statistically significant, there is a higher rate of inhalation injury in the elderly group, 40%, compared to 25% in adults. Previous studies have examined transcriptomic expression profiles and inflammatory profiles in patients with and without inhalation injury and found only minor differences[24]. It may still, however, influence results. There is also evidence to suggest that burn survivors have different inflammatory and stress profiles compared to non-survivors in adult and pediatric studies[3,7]. This study lacked sufficient power to stratify elderly patients based on possible contributing factors such as inhalation injury, co-morbidities, or burn survival, but moving forward we would like to compare the expression profile of these populations.

This study utilized whole blood leukocytes to examine gene expression, but this does not account for specific changes within the leukocyte subpopulations. Leukocytes also provided data on immune-related changes, thus further research using different cell lines is warranted to comment on other areas, such as post-burn metabolism. A larger sample size with characterization within each subpopulation of leukocytes would increase the power of our findings.

It is not clear whether the observed elderly immune dysregulation is the primary driving force behind the poorer outcomes observed in elderly burn patients, or whether it is only a small portion of a global depressed transcriptional response to stress. In this study we did not attempt to make a quantitative argument as to what degree immune dysfunction drives the poorer outcomes associated with elderly burn patients, but did note the presence of significant downregulation of multiple important immune-related pathways. We hypothesize that the identification of specific pathways that are altered in elderly burn patients will serve as a starting point for future studies that examine whether modulation of those pathways affects burn survival and the LD50 for elderly burn patients.

## Conclusions

Elderly patients demonstrate failure to initiate an appropriate inflammatory and stress response during the acute phase post-burn. Pathway analysis revealed downregulation of transcriptomes attributed in the cell signaling and antigen processing in the elderly group. Genes which were observed to be upregulated in elderly patients with high TBSA burn injuries are associated with destruction-related cellular pathways. Our data provide greater insight into the underlying mechanisms contributing to the poorer outcomes observed in elderly burn patients.

## Supporting information

S1 Table Significantly downregulated immune-related gene symbols* for elderly patients based on comparison group 1 (p<0.01, log2fc < (-1)). No named genes were found to be significantly upregulated in elderly patients in this group (p<0.01, log2fc > (1)).
(DOCX)

S2 Table Significantly downregulated immune-related gene symbols* for elderly patients based on comparison group 2 (p<0.01, log2fc < (-1)). (DOCX)

S3 Table Significantly downregulated immune-related gene symbols* for elderly patients based on comparison group 3 (p<0.01, log2fc < (-1)). (DOCX)

S4 Table Significantly upregulated immune-related gene symbols* for elderly patients based on comparison group 2 (p<0.01, log2fc > (1)). (DOCX)

S5 Table Significantly upregulated immune-related gene symbols* for elderly patients based on comparison group 3 (p<0.01, log2fc > (1)). *The full name of each gene symbol can be found on gene databases online, such as at www.genecards.org or https://www.ncbi.nlm.nih.gov/gene/.
(DOCX)
