# Supplementary material for: Genome-wide comparisons of gene expression in adult versus elderly burn patients
Source: PLoS One. 2019 Dec 13;14(12):e0226425. doi: 10.1371/journal.pone.0226425 (PMC6910697; doi:10.1371/journal.pone.0226425)
Supplement: S1 Table — No named genes were found to be significantly upregulated in elderly patients in this group (p<0.01, log2fc > (1)). (DOCX) [file pone.0226425.s001.docx]

**S1 Table. Significantly downregulated immune-related gene symbols* for elderly patients based on comparison group 1 (p<0.01, log2fc < (-1)).** No named genes were found to be significantly upregulated in elderly patients in this group (p<0.01, log2fc > (1)).

| MIS18BP1 |
| --- |
| EGR1 |
| NXPE3 |
| SMC3 |
| MS4A1 |
| LOC101928620/  POU2AF1 |
| BANK1 |
| CCDC88A |
| CTSZ |
| CDCA7L |
| LTN1 |
| TBC1D1 |
| ZBTB20 |
| STRBP |
| LINC00657 |
| C5orf24 |
| CEP97 |
| LEPR/LEPROT |
| LRRN3 |
| SLC30A1 |
| CMTR2 |
| PRR14L |
| NFYB |
| HBS1L |
| C7orf25/PSMA2 |
| MTHFD2L |
| SIKE1 |
| PHAX |
| OGFOD3 |
| FCRL2 |
| ERVK3-1/ZNF8 |
| NEDD1 |
| CLEC5A |
| BLNK |
| METTL2A/METTL2B |
| CLK4 |
| MAP7 |
| HELB |
| CDC27 |
| HAUS6 |
| TMEM263 |
| BRCC3 |
| IQGAP1 |
| BNIP3 |
| SEC22C |
| SBDS/SBDSP1 |
| HSPD1 |
| EEA1 |
| SUGT1 |
| ITGB1 |
| MYO5A |
| KRIT1 |
| CCT2 |
| COG6 |
| AGPS |
| MATR3/SNHG4 |
| RAB30 |
| HINT3 |
| THAP6 |
| LOC145783/ZNF280D |
| ITCH |
| CA5B/CA5BP1 |
| TRAPPC13 |
| CERS6 |
