# Supplementary material for: Genome-wide comparisons of gene expression in adult versus elderly burn patients
Source: PLoS One. 2019 Dec 13;14(12):e0226425. doi: 10.1371/journal.pone.0226425 (PMC6910697; doi:10.1371/journal.pone.0226425)
Supplement: S2 Table — (DOCX) [file pone.0226425.s002.docx]

**S2 Table. Significantly downregulated immune-related gene symbols* for elderly patients based on comparison group 2 (p<0.01, log2fc < (-1)).**

| TET2  BOD1L1 |
| --- |
| MIS18BP1 |
| CCDC186 |
| DHX9 |
| STYX |
| CLIP1 |
| DDX17 |
| NIPBL |
| PTPRC |
| SPEN |
| WNK1 |
| SLC8A1 |
| ESCO1 |
| CCDC88A |
| OSBPL8 |
| CBL |
| SMCHD1 |
| TPR |
| ZBED6 |
| LTN1 |
| SMC3 |
| TBC1D1 |
| CDC27 |
| NDUFS1 |
| THAP6 |
| ZMYM5 |
| PRR14L |
| SECISBP2L |
| REL |
| SMC5 |
| SIKE1 |
| IQGAP1 |
| EEA1 |
| PSME4 |
| SP100 |
| LOC101060691 |
| MIER1 |
| RANBP2 |
| MOB1A |
| IL1A |
| KMT2C |
| ZNF148 |
| HECTD1 |
| CDK5R1 |
| ATRX |
| MAP7 |
| LINC00657 |
| MED13L |
| NUPL1 |
| LRRN3 |
| WASL |
| RAB39B |
| IRF2BP2 |
| MYO5A |
| CCDC125 |
| NFAT5 |
| CEP63 |
| RP11-196G18.24 |
| NAA15 |
| MPZL2 |
| DOCK11 |
| NXPE3 |
| TNRC6B |
| SNORD50A/SNORD50B |
| STRBP |
| ITCH |
| ZBTB20 |
| YTHDC2 |
| SATB1 |
| MLLT3 |
| POLI |
| PNISR |
| LEPR/LEPROT |
| CLK4 |
| AC017002.2 |
| ZNF397 |
| ARRDC3 |
| SF3B1 |
| WLS |
| DENND1B |
| C7orf25/PSMA2 |
| SLX4IP |
| MAN2A1 |
| USP1 |
| IRAK3 |
| CHD2 |
| LOC101928198/ MFAP3L |
| CPEB2 |
| HIST1H3E |
| ARHGAP18 |
| KLHL28 |
| NFYB |
| PHF3 |
| HIST1H2BC |
| INO80D |
| ZNF800 |
| PPIB |
| JADE1 |
| RAPGEF6 |
| LINC01410 |
| CEP350 |
| EHMT1 |
| SAMD9L |
| ST3GAL6 |
| MALAT1 |
| SBDS/SBDSP1 |
| SEC22C |
| SENP6 |
| GKAP1 |
| KIAA2026 |
| HNRNPUL2/ HNRNPUL2-BSCL2 |
| G2E3 |
| ZNF184 |
| HBS1L |
| PRRC2C |
| SLC30A1 |
| SETD2 |
| MTFMT |
| RPAP3 |
| HINT3 |
| EIF3F |
| CHPT1 |
| HIST1H4H |
| ATP7A |
| SOS1 |
| CDK14 |
| ITGA6 |
| HIST1H2AD/HIST1H3A-J |
| REPS2 |
| GON4L |
| RSF1 |
| LINC00674 |
| USP25 |
| BRCC3 |
| SLC35A3 |
| FBXL20 |
| FAM103A1 |
| HMGCR |
| KRAS |
| PPP3R1 |
| NBN |
| IPMK |
| YIPF4 |
| ANKRD44 |
| SNX16 |
| SRPK2 |
| LAMP2 |
| PDCD2 |
| CDC40 |
| MYLIP |
| CPSF2 |
| PRDM2 |
| ZNF268 |
| LPXN |
| LIN7A |
| CWC27 |
| RSBN1L |
| HIST1H2BH |
| GALNT7 |
| RP11-28F1.2 |
| CINP |
| AC092620.2 |
| BTBD7 |
| KRCC1 |
| NPHP3 |
| DIP2B |
| RBM33 |
| STXBP5 |
| THUMPD3 |
| TWISTNB |
| KDM6A |
| HIST1H1C |
| LOC100996668/ZEB1 |
| DNAJC3 |
| AK025288 |
| LARS |
| GAB1 |
| SH3BGRL2 |
| ZFR |
| CPPED1 |
| RNASEL |
| ATP6V1G2-DDX39B/ DDX39B/SNORD84 |
| CSNK1G3 |
| TRMT1L |
| NPL |
| MCTP1 |
| DCAF10 |
| BMP2K |
| CRIPT |
| LPIN2 |
| EXOSC3 |
| CCDC18 |
| PLCL1 |
| ABHD5 |
| AK3 |
| CLEC4A |
| RCBTB1 |
| EVI5 |
| LOC145783/ZNF280D |
| APC |
| SUZ12/SUZ12P1 |
| TMEM55A |
| BDP1 |
| C4orf33 |
| DENR |
| ATXN7 |
| ZNF451 |
| INSC |
| LPGAT1 |
| CPSF6 |
| CDC14B |
| ANGPT1 |
| CHMP5 |
| RAB33B |
| EGLN1 |
| TTC3/TTC3P1 |
| NEK1 |
| DCUN1D1 |
