# Supplementary material for: Genome-wide comparisons of gene expression in adult versus elderly burn patients
Source: PLoS One. 2019 Dec 13;14(12):e0226425. doi: 10.1371/journal.pone.0226425 (PMC6910697; doi:10.1371/journal.pone.0226425)
Supplement: S3 Table — (DOCX) [file pone.0226425.s003.docx]

**S3 Table. Significantly downregulated immune-related gene symbols* for elderly patients based on comparison group 3 (p<0.01, log2fc < (-1)).**

| RETN |
| --- |
| EGR1 |
| SERPINB2 |
| IGHM |
| HP/HPR |
| CEACAM8 |
| EGR2 |
| CLEC5A |
| CD24 |
| CTSZ |
| HP |
| P4HB |
| BPI |
| HSP90AB1 |
| FCRL2 |
| MS4A1 |
| FCRL1 |
| BANK1 |
| SRSF1 |
| MTHFD2L |
| SRD5A1 |
| CDCA7L |
| RABEP2 |
| HLA-DRA |
| TP53I3 |
| BLNK |
| DDOST |
| ERVK3-1/ZNF8 |
| LILRA5 |
| CAPN3 |
| SDHC |
| COG6 |
| MS4A4A |
| FAM20A |
| TRMT6 |
| KBTBD6 |
| TMPO |
| FGD3 |
| KRIT1 |
| PLA2G4A |
| VSTM1 |
| P2RX5 |
| LGALS1 |
| CMTM4 |
| YBX1 |
| BAX |
| CMTR2 |
| NIPSNAP3A |
| ALYREF |
| TMEM256 |
| CD200 |
| YIF1B |
| ATP9B |
| LAMC1 |
| TCL1A |
| FOSB |
| HSPA8/SNORD14C,D |
| MPDU1 |
| RPL8 |
| SNHG5/SNORD50A,B |
| SAMHD1 |
| RPL18A/SNORA68 |
| SUCLA2 |
| TMCO3 |
| CFL1 |
| THBS1 |
| CA4 |
| NAGK |
| SUGT1 |
| FCN1 |
| METTL2A,B |
| PI4KA |
| KDELR1 |
| COA3 |
| ABCC10 |
| CCT3 |
| MIR4691 MIR7113 NDUFS8 |
| RPL18 |
| CDC37 |
| AP2S1 |
| VAMP4 |
| LOC101928429 |
| CLPTM1L |
| FAM134C |
| SCAND1 |
| CHCHD7 |
| NUDT4/NUDT4P1/ NUDT4P2 |
| CEP97 |
| IDH3A |
| MAD1L1 |
| TMEM173 |
| PECR |
| RNF14 |
| PTX3 |
| TOR1AIP1 |
| KLF4 |
| BPNT1 |
| KMO |
| ACTB |
| WDR11 |
| ZNF780A |
| SLC30A1 |
| ZUFSP |
| CMAS |
| POLE4 |
| OGT |
| TMEM14B |
| ANKRD55 |
| MYOF |
| SS18 |
| PSMD3 |
| PSAT1 |
| LARS |
| LOC100049716 |
| GGH |
| BST2 |
| YTHDC2 |
| MFSD10 |
| SLX1A-SULT1A3/ SLX1B-SULT1A4/SULT1A3/SULT1A4 |
| FAM118B |
| TRAPPC13 |
| DYNC1I2 |
| UBE4B |
| FAM126A |
| FAM160A2 |
| MT1X |
| LYZ |
| FAM175B |
| SULT1A1 |
| UROD |
| TIMM17A |
| NDUFAF6 |
| TM2D1 |
| LOC439994/LOC642361 |
| TFCP2 |
| CSTF2T |
| FCAR |
| CERS6 |
| SNX17 |
| SMIM7 |
| UBE3C |
| KDELR2 |
| GPX1 |
| MCFD2 |
| ADORA2B |
| CORO2A |
| MRPL43 |
| GNS |
| MMP9 |
| GIMAP1 |
| CRLS1 |
| TMX1 |
| TCAIM |
| SAT2 |
| LILRA3 |
| HIPK2 |
| RPS16 |
| TMOD3 |
| DHCR7 |
| GPR97 |
| SPTLC2 |
| ELOVL1/MIR6734 |
| ZSWIM7 |
| IER2 |
| TM9SF4 |
| TGOLN2 |
| IPO11/IPO11-LRRC70 |
| DPP3 |
| RP11-4O1.2 |
| POLR2L |
| SIKE1 |
| RNF7 |
| TRMT1L |
| CHCHD5 |
| PFDN5 |
| DSTN |
| CHTOP |
| TMEM87B |
| COMMD10 |
| CCT2 |
| CES1 |
| SLC25A6 |
| DNAJC5 |
| AGPS |
| PDHA1 |
| PSMD2 |
| DDX39A |
| UEVLD |
| ARGLU1 |
| ANXA5 |
| UXS1 |
| NELFB |
| BABAM1 |
| SLC38A6 |
| LDLR |
| VPS45 |
| POP5 |
| SIAE |
| MAVS |
| CNPY2 |
| UFC1 |
| PAIP1 |
| PFDN2 |
| SLC35B4 |
| HDDC3 |
| HYPK/MIR1282/SERF2 |
| ID2 |
| PDIA6 |
| RANBP9 |
| PTPN22 |
| RPE |
| NDUFS7 |
| TCF4 |
| ILF3 |
| TBCK |
| CHML |
| ELMO2 |
| FBXO3 |
| CCND3 |
| ATOX1 |
| XPOT |
| FOXK1 |
| CYBB |
| SFPQ |
| MSH2 |
| COX8A |
| MIR6824/SLC26A6 |
| LARP4 |
| AGA |
| C11orf71 |
| TCTEX1D2 |
| HNRNPM |
| COPZ1 |
| MRPS28 |
| UPF3A |
| MRPL42 |
| TSPAN3 |
| RBM6 |
| MESDC1 |
| HYOU1 |
| SMARCA2 |
| ANXA1 |
| YWHAG |
| ORMDL2 |
| SNORA29/TCP1 |
| SNAP23 |
| NMT1 |
| SQLE |
| BTBD3 |
| PSME3 |
| LSS |
| SEC61A1 |
| MAPKAP1 |
| PSMB1 |
| SEPW1 |
| SLC25A40 |
