# Supplementary material for: Genome-wide comparisons of gene expression in adult versus elderly burn patients
Source: PLoS One. 2019 Dec 13;14(12):e0226425. doi: 10.1371/journal.pone.0226425 (PMC6910697; doi:10.1371/journal.pone.0226425)
Supplement: S4 Table — (DOCX) [file pone.0226425.s004.docx]

|  |
| --- |

**S4 Table. Significantly upregulated immune-related gene symbols* for elderly patients based on comparison group 2 (p<0.01, log2fc > (1)).**

| TMTC1 |
| --- |
| TRIB1 |
| JUND |
| ELMO2 |
| RHBDD2 |
| RXRB |
| RHBDF2 |
| SIRPAP1 |
| KLF16 |
| SZRD1 |
| CDKN1C |
| DCAF15 |
| FURIN |
| EHBP1L1 |
| TRPV2 |
| MIR4745/PTBP1 |
| GPR132 |
| LRRC25 |
| TBCB |
| SPON2 |
| GSDMD |
| TTYH3 |
| CORO7 |
| PHF1 |
| COL6A2 |
| ARFGAP1 |
| CDK16 |
| RAB29 |
| RANGAP1 |
| CHD3 |
| CDK14 |
| VARS |
| MOSPD3 |
| HMOX1 |
| SRRM2 |
| METRNL |
| SSH1 |
| NPIPA1/2/3/5/ NPIPA 7/8  PKD1P1 |
|  |
| ARFGAP2 |
| AP2A1 |
| TPP1 |
| PFKL |
| GBF1 |
| PTGDS |
| GYPC |
| ACVR1B |
| GNS  ACAP3 |
