# Supplementary material for: Genome-wide comparisons of gene expression in adult versus elderly burn patients
Source: PLoS One. 2019 Dec 13;14(12):e0226425. doi: 10.1371/journal.pone.0226425 (PMC6910697; doi:10.1371/journal.pone.0226425)
Supplement: S5 Table — *The full name of each gene symbol can be found on gene databases online, such as at www.genecards.org or https://www.ncbi.nlm.nih.gov/gene/. (DOCX) [file pone.0226425.s005.docx]

**S5 Table. Significantly upregulated immune-related gene symbols* for elderly patients based on comparison group 3 (p<0.01, log2fc > (1)).**

| RSAD2 |
| --- |
| MALAT1 |
| LOC100505478 |
| C19orf26 |
| CC2D2B |
| LINC00173 |
| MAP3K12 |
| HOTTIP |
| C10orf25 |
| HCG27 |
| NKX1-1 |
| C20orf203 |
| DOCK4 |
| GALNT8/KCNA6 |
| STAT1 |
| LOC93444 |
| DISC1 |
| PLIN5 |
| SOD3 |
| FUT6 |
| PP12719 |
| EDA |
